# Predicting Cybersecurity Threats in Critical Infrastructure for Industry 4.0: A Proactive Approach Based on Attacker Motivations

**DOI:** 10.3390/s23094539

**Published:** 2023-05-06

**Authors:** Adel Alqudhaibi, Majed Albarrak, Abdulmohsan Aloseel, Sandeep Jagtap, Konstantinos Salonitis

**Affiliations:** 1 School of Aerospace Transport and Manufacturing (SATM), Cranfield University, Cranfield MK43 0AL, UKk.salonitis@cranfield.ac.uk (K.S.); 2 School of Information Studies, Syracuse University, Syracuse, NY 13244, USA

**Keywords:** critical infrastructure, cyberattack, cybersecurity, cyberthreats, cyber-physical security, ICS security, industry 4.0, motivation, predict, SCADA security

## Abstract

In Industry 4.0, manufacturing and critical systems require high levels of flexibility and resilience for dynamic outcomes. Industrial Control Systems (ICS), specifically Supervisory Control and Data Acquisition (SCADA) systems, are commonly used for operation and control of Critical Infrastructure (CI). However, due to the lack of security controls, standards, and proactive security measures in the design of these systems, they have security risks and vulnerabilities. Therefore, efficient and effective security solutions are needed to secure the conjunction between CI and I4.0 applications. This paper predicts potential cyberattacks and threats against CI systems by considering attacker motivations and using machine learning models. The approach presents a novel cybersecurity prediction technique that forecasts potential attack methods, depending on specific CI and attacker motivations. The proposed model’s accuracy in terms of False Positive Rate (FPR) reached 66% with the trained and test datasets. This proactive approach predicts potential attack methods based on specific CI and attacker motivations, and doubling the trained data sets will improve the accuracy of the proposed model in the future.

## 1. Introduction

In today’s world, technology plays a crucial role in the smooth and continuous flow of information and communication, which is essential for the success of any business [1]. The evolution and constant improvement of technology have brought about the industry 4.0 revolution, which has transformed various sectors, including technology, industries, and infrastructure [2]. The rapid digitalization worldwide, powered by advancements in Information and Communication Technology (ICT), has opened up enormous possibilities for growth [3]. However, according to Gartner, by 2025, 30% of Critical Infrastructure (CI) businesses will experience a security breach, which may result in shutting down mission-critical cyber-physical systems (CPS). Gartner also predicts that, by 2025, attackers will have effectively weaponized a CI’s CPS, leading to harm or death of individuals [4,5]. Understanding the cost of a data leak involves considering various factors, such as a company’s location and activity, the sensitivity of stolen data, and the magnitude of the security breach, which could result in unforeseen consequences and damages.

**Figure 1:**
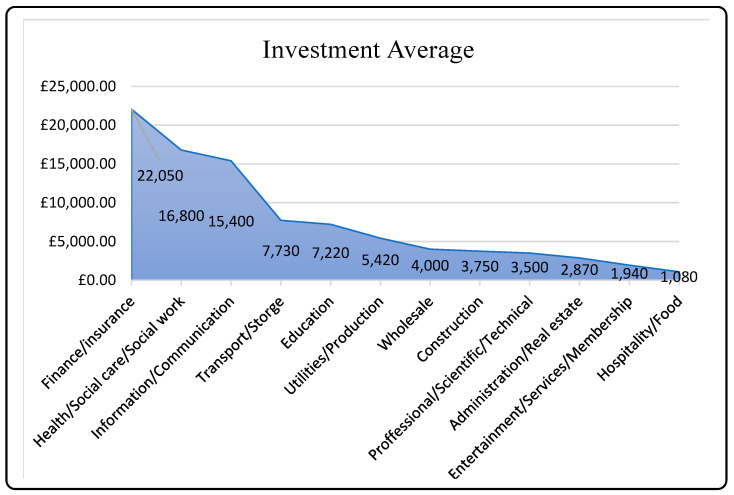
Average investment in cybersecurity by various businesses.

There has been an increase in cyberattacks across all sectors [6,7], with hackers stealing sensitive data, including personal information of consumers and workers, and intellectual property of organizations. Breaches can have a negative impact on stakeholder confidence and trust, depending on the severity of the attack and the extent of its public exposure. The food and hospitality industries are the least likely to prioritize cybersecurity, according to the Cybersecurity Breaches Survey 2022 by the Department for Digital, Culture, Media & Sport [8]. This is because they are less likely to use security measures, such as two-step verification, and less likely to report having up-to-date malware protection, password policies, or network firewalls [8]. In 2021, the food and beverage and construction sectors in the UK treated cybersecurity as a lower business concern compared to other businesses, with only 62% and 64%, respectively, considering it to be of high importance, versus 77% overall. The graph depicted in Figure 1 shows the amount of money spent on cybersecurity by CI sectors in the UK and the average investment made in cybersecurity by various business sectors in 2019 [9,10].

**Figure 2:**
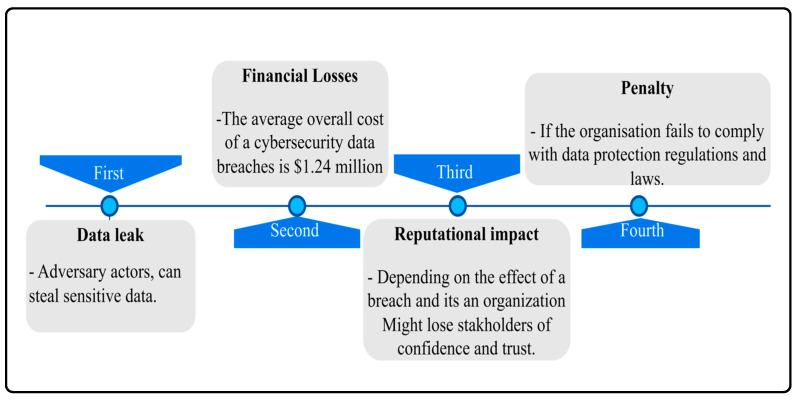
Cybersecurity breach impact when having an absence of cyber measures.

The Centre for Strategic and International Studies (CSIS) estimates that cybercrime costs the global economy more than $400 billion every year [11]. Additionally, a report released by McAfee in December 2020 reveals that the global cost of cybercrime losses has surpassed $1 trillion, increasing by over 50% since 2018 [12]. Furthermore, IBM’s 2020 report states that the average cost of a cybersecurity data breach is $1.24 million [13]. These statistics demonstrate that no industry is immune to cybercrime’s growing impact, emphasizing the need to prioritize security measures, tools, and solutions to address the most critical vulnerabilities in the infrastructure. Predicting cyberattacks is crucial for protecting CI systems from potential cyber threats. A cyberattack can affect an organization in four different ways, including data leaks involving sensitive data, personal data of consumers, and an organization’s intellectual property, as well as financial losses, reputational damage, and penalties resulting from failing to comply with data protection regulations established by government agencies. Figure 2 illustrates these consequences [14].

Governments and organizations are encouraged to adopt advanced security solutions based on Artificial Intelligence (AI) and Machine Learning (ML) techniques to predict cyberattacks on Critical Infrastructure (CI). CI plays a vital role in countries worldwide and is essential to maintain essential societal functions, public security, and economic sustainability. Recent natural disasters, security threats, and political issues require governments to establish policies and standards that emphasize resilience and stability in the CI sector [15]. In response to the high consequences of technological catastrophes and capital investments, decision-makers prepare CI for any potential threat scenario. Cyberattacks can affect CI sectors in various ways, and cyber analysts can use ML to predict and mitigate the danger by identifying patterns discovered in previously detected malware incidents [16]. The definition of CI sectors may vary slightly between different nations. Generally, CI refers to systems, assets, or parts of them that are crucial for maintaining essential societal functions, public security, and economic sustainability. The destruction of these functions would have severe consequences for the country as a whole [17].

The National Institute of Standards and Technology (NIST) offers a guide for organizations to manage their internal and external cybersecurity risks. This guide consolidates industry best practices and standards to combat cybercrime. Although it was initially intended for the Critical Infrastructure (CI) sector, it has now extended to other industries, as well. The NIST guide consists of five functions: identify, protect, detect, respond, and recover [18]. CI systems, such as those that manage power grids, water and wastewater treatment, and oil production, are interconnected to form the energy grid [19]. A hacktivist group caused panic by taking down the US electrical grid during a particularly harsh winter. This resulted in bank and hospital closures, power outages for millions of homes and businesses, communication disruptions, and grounded air traffic. This situation seems to have been a disaster, but it was a practical threat [19]. Therefore, it is crucial to be proactive in addressing cybersecurity risks, now more than ever.

The objective of this research is to predict cybersecurity threats and attacks on Critical Infrastructure (CI) systems by utilizing ML algorithms. The research aims to identify and highlight potential attacks that result from a lack of cybersecurity measures and establish a proactive security approach to safeguard CI systems against cyber threats. Additionally, the study seeks to assess the significance of cybersecurity for CI systems and obtain a better understanding of the current state of cybersecurity in the CI and its readiness to combat cyber threats.

To detect cyberattacks using prediction models, three main elements are required: ML algorithms, datasets, and characteristics of the trained data [20,21]. Cyber analysts can use ML to predict and mitigate the threat by identifying patterns discovered in previously detected malware [22]. Cybersecurity procedures follow a lifecycle model of prediction, detection, prevention, and reaction, similar to other IT processes [23]. Organizations must take all preventative measures during the prediction phase to identify potential attackers, their motives, and the techniques they plan to use. This involves gathering information about cyber threats and performing risk analysis [24]. Following a standardized methodology, such as ISO 31000, is crucial, and procedures should include defining the scope and objectives for risk management, conducting a risk assessment, and generating appropriate risk mitigation plans [25]. The research proposes a new approach to predict cyberattacks in CI based on ML algorithms and attackers’ motivations. The study tests various ML algorithms and aims to maximize the use of real datasets to improve prediction accuracy. The prediction model can be customized, depending on the nature of the CI and attacker motivations. 

## 2. The Literature Review

Numerous approaches have been developed for detecting and classifying cybersecurity threats, such as malware and malicious code, many of which involve ML and text analysis [26]. ML techniques have proven to be beneficial in detecting malware, according to various related studies. For example, Suh-Lee et al. (2017) used data mining, natural language processing, ML, and categorization to detect security concerns by extracting relevant data from different unstructured log messages [27]. Kakavand et al. (2014) developed the Text Mining-Based Anomaly Detection (TMAD) model to identify HTTP attacks in network traffic, which uses term frequency, inverse document frequency, TF-IDF techniques, and n-gram text categorization [28]. Norouzi et al. (2016) developed several classification approaches to detect malware programs based on the characteristics and behaviors of each program [29]. Fan et al. (2015) used hooking techniques and ML algorithms, such as I Bayesian, Decision Tree, and Support Vector Machine (SVM), for the categorization process [30]. Hellal and Romdhane (2016) proposed an approach that combines graph mining methods and static analysis and the Minimal Contrast Frequent Subgraph Miner (MCFSM) algorithm to detect minimally discriminative malicious behavioral patterns [31]. However, it is essential to note that these studies did not consider attacker motivations.

A data mining approach has been established to identify actionable knowledge of internet threats [32]. This approach involves two stages. The first stage involves unsupervised clique-based clustering on complex patterns from the data to identify groups of similar patterns based on a single property. In the second stage, different sets of cliques are combined to create ‘concepts’ with a common similarity pattern that accurately describes the real-world phenomenon to provide a more in-depth understanding of emerging Internet threats and attacks. The researchers validated their approach using a large dataset gathered via a globally distributed honeynet. Another study proposed a correlation intrusion attack prediction system with two key components: the attack scenario extraction algorithm (ASEA) and plan recognition [33]. ASEA is used to mine the alert stream for attack scenarios, while plan recognition employs a hidden Markov model to predict the next infiltration attempt and is evaluated using the DARPA 2000 data set. Additionally, a network security prediction approach, incorporating dynamic backpropagation neural with covariance, has been developed [34]. The researchers used the situation assessment model to collect the situation sequences, used them as input training data, and applied self-learning dynamic adjustment of the values of chosen parameters. However, these methods did not consider the attacker’s motivation factor.

A proposed intrusion forecasting system consists of three modules: data collection, data analysis, and reporting [35]. The data analysis system integrates three techniques for predicting DDoS attacks on the DARPA 2000-specific data set, namely, time-series analysis, probabilistic modelling, and data mining. Combining multiple forecasting approaches can significantly reduce the false alarm rate. However, existing methods have limitations in predicting cyber-attacks based on attacker motivations. There is still some uncertainty regarding the effects of I4.0 on cybersecurity, and many organizations are unaware of the challenges that come with integrating the I4.0 framework [36]. Despite this, a suitable manufacturing model and planning target roadmaps can help enterprises prepare for the changes associated with I4.0 [37]. This paper proposes a security prediction model using ML techniques to enhance CI cybersecurity and increase its resilience and stability.

## 3. Predicting Cyberattacks on Critical Infrastructure (PCCI) Approach

To introduce the approach to PCCIs, it is important to consider the relevant components, including an understanding of the nature of CIs and their categories, as well as the role of ML algorithms in predicting cyberattacks on these infrastructures. By doing so, a theoretical concept of the prediction mechanism can be developed and applied in practice. This section has been divided into four subsections: (1) CI categories, (2) the role of ML algorithms in prediction, (3) the theoretical concept of a prediction mechanism based on PCCI, and (4) the practical implementation of the proposed approach.

### 3.1. Critical Infrastructure Categories

**Figure 3:**
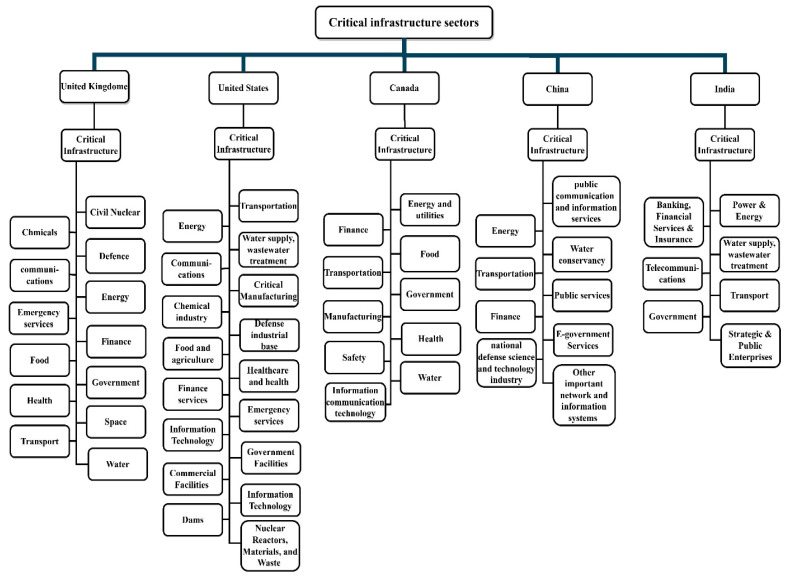
Sectors of designated CI across several countries.

Different countries have different ways of categorizing critical infrastructure (CI) sectors, which are crucial for the functioning of their economy, society, security, and services. For instance, in the US, the National Infrastructure Protection Plan (NIPP), in 2013, categorized CI into 16 sectors, such as energy, water and wastewater systems, healthcare and public health, information technology, transportation systems, etc. [38]. Similarly, the European Union classified CIs into 13 categories, including energy, ICT, water, food, health, financial, and public safety, among others. These categorizations help to sort the critical infrastructure based on their impacts and effects. Figure 3 depicts the categorization of CI in various countries [17].

**Figure 4:**
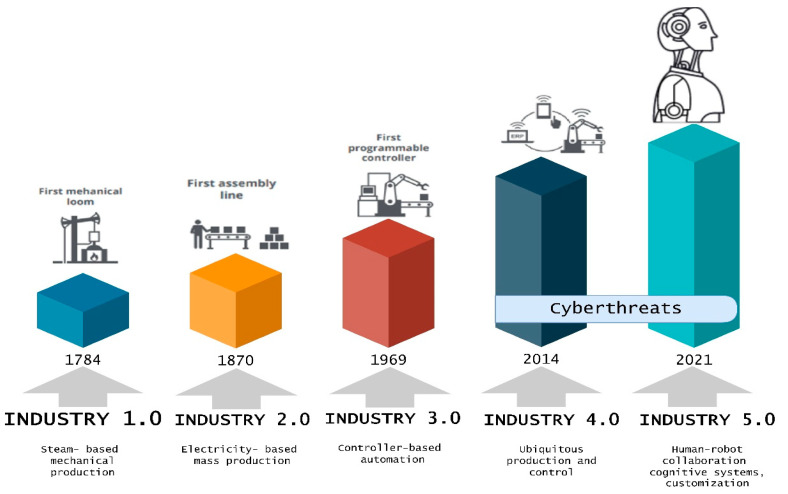
Cyber and physical threats for each industrial revolution.

The digitalized integration and intelligent industrial engineering of I4.0 have transformed the manufacturing paradigm. As a result, cyber threats have intensified and expanded beyond those observed in the digital network, as CI systems become increasingly networked. The manufacturing lines could be subject to attacks that lead to monetary loss, worker safety issues, or poor product quality. Moreover, the networks could also be vulnerable to attacks, leading to misuse or shutdowns. The evolution of industrial revolution is posing new threats, as illustrated in Figure 4.

The use of advanced technologies, such as smart products, networks, logistics, and the Internet of Things, has drastically transformed existing value chains and led to the development of new business models, making smart factories an essential element of future infrastructure [39]. This infrastructure generates numerous benefits, including new business models and the creation of value, particularly for traditional manufacturing companies [40]. One of the most significant disruptions in Industry 4.0 is the increasing value and significance of data, making it a valuable commodity. Conventional manufacturing companies need to change their approach to managing vast amounts of data, which is a significant obstacle [41]. The use of programmable production technology combined with machine flexibility, such as flexible grip hooks, has several advantages, including individualized customization, more dynamic resource allocation, quicker changeover times, and decreased production complexity. This makes production processes quicker, cheaper, easier, and more diverse. Businesses can benefit from Industry 4.0 in several ways, such as reduced labor costs, simplified business processes, increased transparency in logistics, and enhanced productivity and revenue. Virtual and augmented prototyping allows for interactive exploration of all product functionalities, providing a thorough understanding of product features and benefits, which play a vital role in enhancing productivity and revenue, thus stimulating economic growth [42].

Manipulating industry machines, altering product data, and other important information can make manufacturing infrastructure unsafe for sale. For example, tampering with serial numbers can result in loss of company brand reliability and business. Personal information of customers can be stolen, resulting in a breach of the GDPR privacy and security law, which could lead to financial penalties. Cyberattacks can also disrupt the supply chains and storage of CI, resulting in the halt of the system’s operation. One of the main challenges in smart CI is protecting it from potential cyberattacks. The impact of cybersecurity threats could damage reputation, finances, data, and safety.

### 3.2. The Role of Machine Learning Algorithms in Attack Prediction

**Figure 5:**
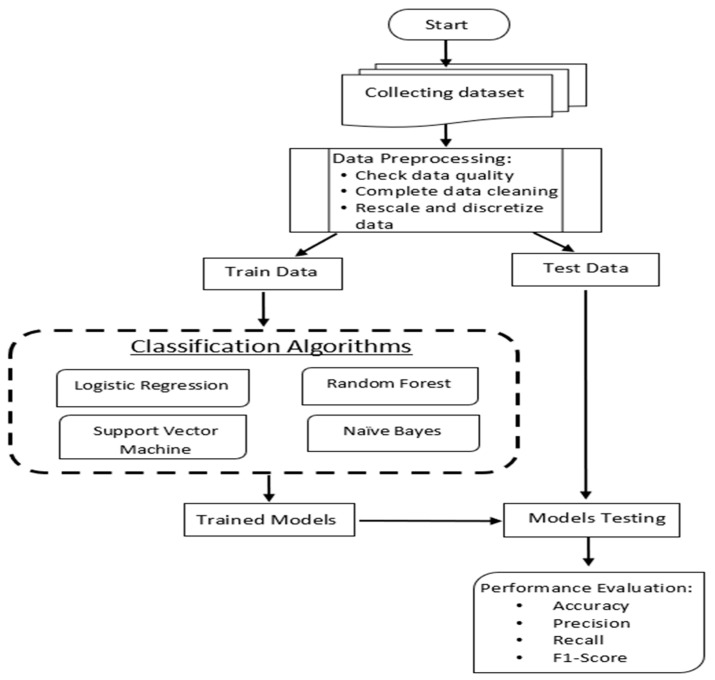
Prediction mechanism of the PCCI.

As a proactive step, predicting cybersecurity threats will prepare the target infrastructure for protecting their assets and reputation from these threats. However, predicting cyberattacks is difficult regarding less information and data open to public. In the PCCI approach, we develop a predicting model based on the ML algorithm based on the open-source dataset. Figure 5 explains the ML method steps, which are implemented in the PCCI approach.

The measures of recall, precision, and F1-score are formulated to evaluate the performance of each model and to assess the classifiers performance. The definition for these metrics is as follows.

Although accuracy provides a general measure of a classifier’s performance, accuracy alone is insufficient and must be combined with the concepts of recall and precision.

(1) $$\mathrm{Recall}=\frac{\mathrm{True}\;\mathrm{Positives}}{\mathrm{True}\;\mathrm{Positives}+\mathrm{False}\;\mathrm{Negatives}}$$

The accuracy of this model’s performance for positive classes can also be evaluated by using recall. To define the percentage of positive predictions that were correct, we used precision, which is defined as:

(2) $$\;\mathrm{Precision}=\frac{\mathrm{True}\;\mathrm{Positives}}{\mathrm{True}\;\mathrm{Positives}+\mathrm{False}\;\mathrm{Positives}}$$

In addition to precision and recall, the F1-score is also used. F1-score is effective and indicates the balance between recall and precision. In order to balance precision and recall, the F1-score is employed to evaluate the test’s accuracy.

(3) $$\;F1\;\mathrm{score}=2\;\frac{\mathrm{Precision}\cdot \mathrm{Recall}}{\mathrm{Precision}+\mathrm{Recall}}$$

### 3.3. The Theoretical Concept of the Prediction Mechanism Based on PCCI Approach

**Figure 6:**
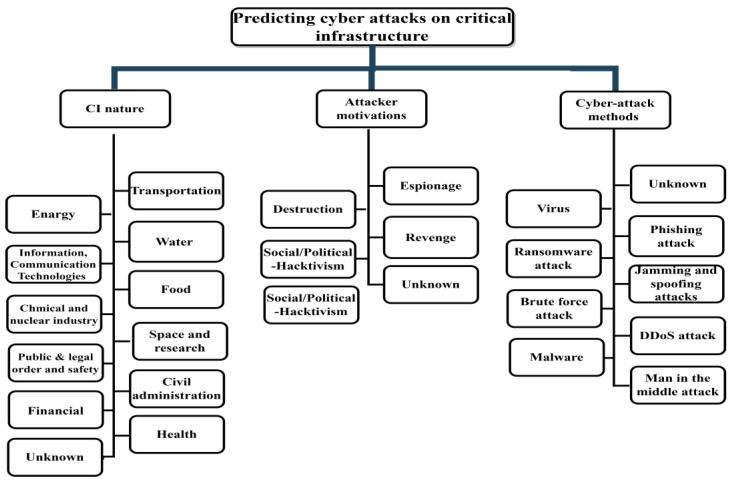
PCCI approach main data elements.

Regarding the prediction mechanism of the PCCI approach, first, realize the nature of the CI related to their attacks for contingency planning to increase security. Then, determine the attacker’s motives through threat intelligence or by understanding the primary motivations that guide cyberattacks against CI facilities or governments. These motivations can identify where critical assets are at risk. As a result, they more efficiently and effectively address determined risks. Security engineers and specialists in this sector must adopt security countermeasures to detect, prevent, and deter these attacks. For instance, knowing the nature of the services offered by financial institutions and banks incites the attacker to steal money and do illegal harm to capital owners and their enterprises. The necessary phases of the PCCI approach are outlined in the following steps, and Figure 6 illustrates the main elements of the PCCI approach:

- Step 1: Determine the CI’s functioning, including the infrastructure’s service and the system’s hardware and software components.
- Step 2: Determine the motives for the attack. There may be multiple reasons at play; the reasons may vary in intensity.
- Step 3: Linking the first and second steps, the cybersecurity professional can predict potential cyberattacks according to CI targets and attacker motivations.
- Step 4: Implement the required security measures to avoid cyberattacks as a preventative security precaution. By improving the understanding of recorded cyberattacks on various CI systems and categorizing the attacks to develop a predictive model of cyberattacks based on ML, this proposed approach, in its initial form, can be taken further, making the prediction of attacks more sophisticated and efficient. It takes time and cooperation across cybersecurity agencies to construct and update vast datasets and databases to gather the required cybersecurity data.

### 3.4. Practical Implementation of The PCCI Approach

To effectively apply ML principles, datasets must be properly normalized; data collected from different resources [43,44] contain recent cybersecurity incidents. In the current phase, the features are the attacked organization’s infrastructure, the incidents’ data, and the motivation that stimulated the attack. The proposed approach is an attempt to predict the methods that have been used in such attacks. In this work, after collecting more than 400 incidents, data cleansing for the raw data was performed, as well as exploring the data to obtain more insight into the incident’s patterns. For the model training, we have split our dataset into testing and training subsets. The ratio used for our experiments was 80% for training and 20% for testing. This has resulted in a total of 320 entries for training and 80 for testing. The testing part will be utilized to evaluate the performance of our trained model. Whereas the training part will be used to train the algorithms. We have predicted cyberattacks from the testing part of our dataset after training the chosen algorithms, which were used in our models. Then, the predictions were used to assess the performance of the model using the performance metrics of accuracy, recall, precision, and F1-score. Finally, this task is performed by Algorithm 1, which summarizes the proposed approach:
**Algorithm 1:** Pseudocode of proposed approach.  Input: Data Output: Classification results**Begin**  Dataset collection and scraping  Dataset preparation and preprocessing  Data cleaning  Data splitting  Model building  Classification results  Evaluation of results**End**

**Figure 7:**
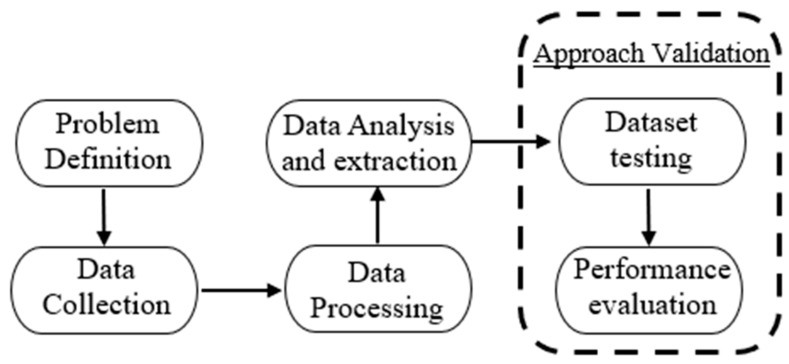
Data processing stages.

Six stages comprise the third phase, which begins with problem definition and ends with approach validation. These six stages are summarized in Figure 7.

As a first step to practically implement the PCCI approach, the CIs have been divided into twelve sectors, depending on the Commission of the European Union, which are listed in the following code [18]. In the ML context, the dataset features of the datasets have been converted to numerical values to perform the training model to find the prediction percentage accuracy tuning faster and to obtain more accurate results. Algorithm 2 has been executed to complete this task:
**Algorithm 2:** In [7]: convert Infrastructure Label. **Begin**  df[“Infrs_No”] = df[‘Infrstructure’]  df[“Infrs_No”] = df[‘Infrs_No’].replace(to_replace =“Unknown”, value = “0”)  df[“Infrs_No”] = df[‘Infrs_No’].replace(to_replace =“Energy”, value = “1”)  df[“Infrs_No”] = df[‘Infrs_No’].replace(to_replace =“Information, ICT”, value = “2”)  df[“Infrs_No”] = df[‘Infrs_No’].replace(to_replace =“Water”, value = “3”)  df[“Infrs_No”] = df[‘Infrs_No’].replace(to_replace =“Food”, value = “4”)  df[“Infrs_No”] = df[‘Infrs_No’].replace(to_replace =“Chemical and nuclear industry”, value = “5”)  df[“Infrs_No”] = df[‘Infrs_No’].replace(to_replace =“Space and research”, value = “6”)  df[“Infrs_No”] = df[‘Infrs_No’].replace(to_replace =“Transport”, value = “7”)  df[“Infrs_No”] = df[‘Infrs_No’].replace(to_replace =“Public & legal order and safety”, value = “8”)  df[“Infrs_No”] = df[‘Infrs_No’].replace(to_replace =“Chemical and nuclear industry”, value = “9”)  df[“Infrs_No”] = df[‘Infrs_No’].replace(to_replace =“Civil administration”, value = “10”)  df[“Infrs_No”] = df[‘Infrs_No’].replace(to_replace =“Health”, value = “11”)  df[“Infrs_No”] = df[‘Infrs_No’].replace(to_replace =“Financial”, value = “12”)**End**

Before building our model, a histogram graph for each numeric variable is plotted to give us a clearer image of the distribution of each input variable, and to implement this technique, the following command has been executed practically, as shown in Algorithm 3:
**Algorithm 3:** Plotting a histogram chart to obtain a clearer picture of the relationship of the input.**Begin**  import pylab as pl  df_num.hist(bins = 30, figsize = (9.9))  pl.suptitle(“Histogram for each numeric input variable”)  plt.savefig(‘df_num_hist’)  plt.show()**End**

**Figure 8:**
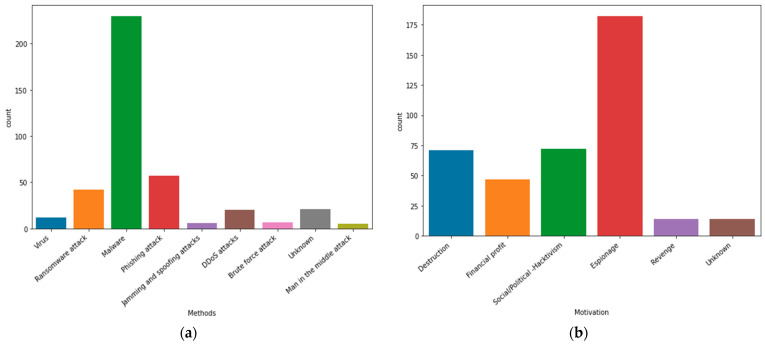
(**a**) The cyberattacks methods implemented in this approach. (**b**) Describe attackers’ motives that are used in this approach.

Attackers’ motivations have been identified as follows: destruction, financial profit, social/political (hacktivism), espionage, revenge, and unknown. Besides that, the attack methods have been determined as follows: virus, ransomware attack, phishing attack, jamming and spoofing attacks, DDoS attack, brute force attack, malware, man in the middle attack, and unknown. Additionally, the following charts illustrate the results in terms of cyberattack methods and attackers’ motives, which are implemented in the PCCI approach. Figure 8a,b show the count of attackers’ motivations and cyberattacks methods.

For the final step, the datasets were loaded for the training session using a logistic regression and fine tree classifier classification learner. For example, running the following python code generates the prediction model (Algorithm 4).
**Algorithm 4:** Python code generate the prediction model.**Begin**  from sklearn.svm import SVC  svclassifier = SVC(kernel = ‘linear’)  svclassifier.fit(X_train, y_train)  y_pred = svclassifier.predict(X_test)  from sklearn.metrics import classification_report, confusion_matrix  print(confusion_matrix(y_test,y_pred))  print(classification_report(y_test,y_pred))**End**

## 4. The Prediction Results of the PCCI Approach and Discussion

In this research, we proposed a prediction approach that facilitates the detection of future cyberattacks method based on previous cyber incidents. This methodological approach bears a significant impact on CIs that choose to implement advanced security models to enhance their services. Meanwhile, a classified dataset should be used in this security model, as most governments and organizations classify cyber incidents as secret information [45]. We determine twelve CI sectors based on the green paper on a European program for CI protection [20]. Any organizations with a sensitive environment must protect their assets and data by using a proactive security model. The proposed security model gives predictions for future attack methods by using a trusted dataset. However, most governments and organizations classify cyber incidents as a piece of secret information. This section discusses several of our security model benefits to CIs and security countermeasures.

The responsible CI security and protection team is the first to benefit from the PCCI approach’s deployment to predict cyberattack methods. Forecasting the cyberattack methods will help the CI security team to defend and protect their systems better, conduct urgent cybersecurity awareness and training, and match the needs of a new project plan and the decision maker’s budget preferences. Therefore, a proactive cyber security approach will improve the cyber security level for CIs and ensure that CI assets and systems are protected. In addition, being proactive and seeking a new source of information and attack tactics will emphasize the security of CIs assets and data. Predicting cyberattack methods is an advanced step in a cyber security solution, such as threat intelligence. Another aspect is to improve CI systems’ security, which is greatly enhanced by deploying and predicting cyberattack methods. CI systems security can also be promoted by training and cyber exercises for cyber security practitioners. Prediction, awareness, training, and cyber exercise are essential to improve CI cybersecurity towards a resilient and vigilant organization.

The result of a prediction should be a future actuality. Until that future day arrives, no one can provide the correct responses. PCCI prediction results (Table 1) will be presented in this section after implementing the training dataset on Support Vector Machine (SVM) models, which are logistic regression and fine tree classifier, and we found the highest accuracy with linear and poly models.

**Figure 9:**
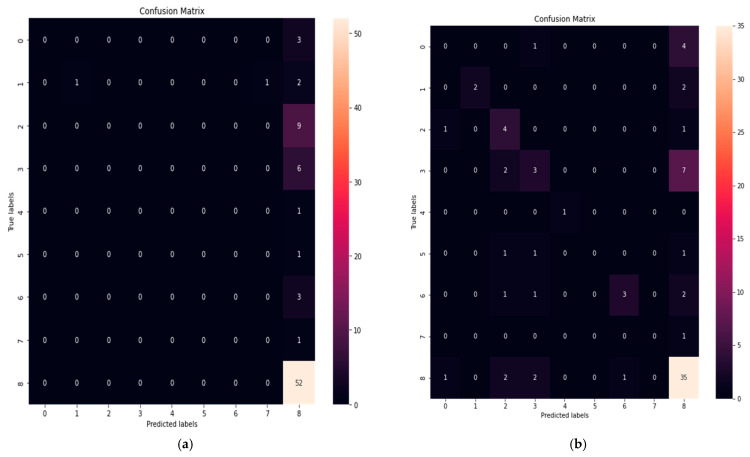

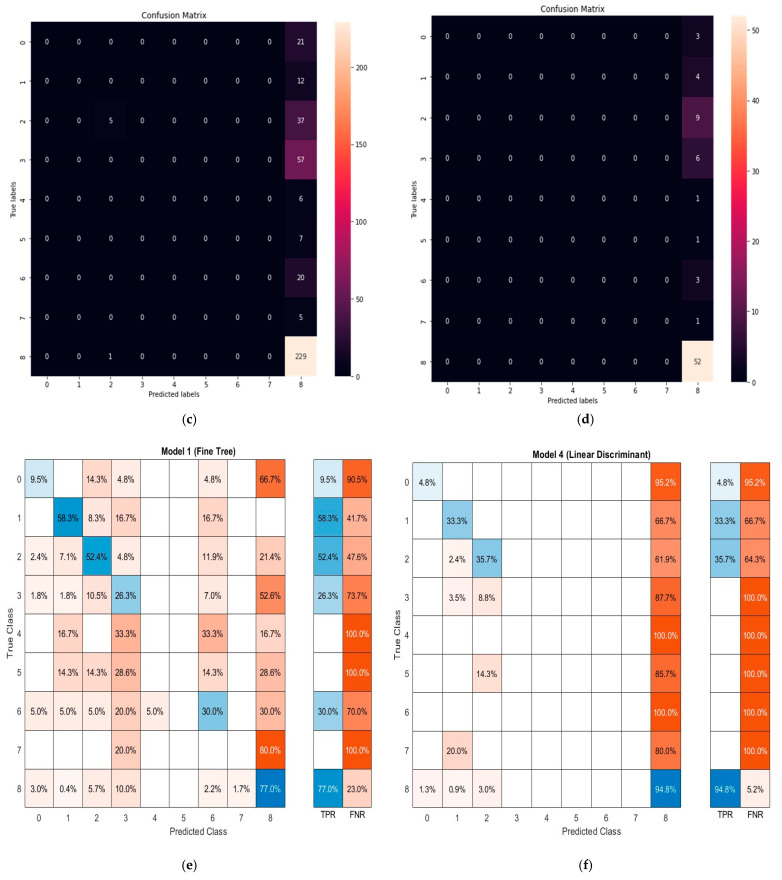

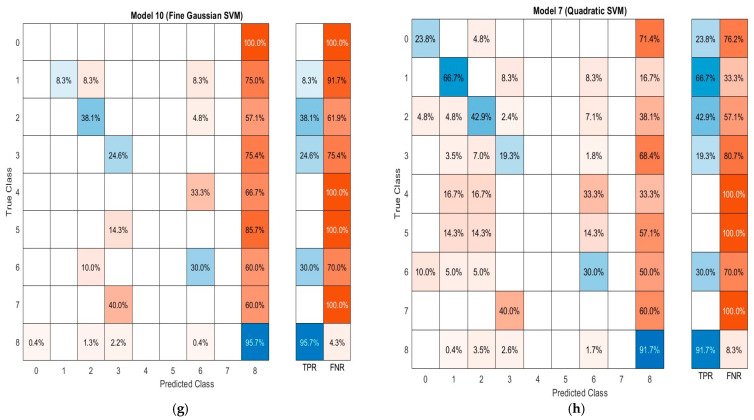
(**a**) Linear model; (**b**) Decision Tree Classifier model; (**c**) Logistic Regression model; (**d**) Poly model; (**e**) Fine Tree model; (**f**) Linear Discrimination model; (**g**) Fine Gaussian SVM model; (**h**) Quadratic SVM model.

A classification problem’s prediction outcomes are summarised in a confusion matrix. The confusion matrix demonstrates how the classification model makes predictions while being confused. Count values are used to describe the number of accurate and inaccurate predictions for each class, which is the confusion matrix’s secret. The following, Figure 9a–h, show the confusion matrix for all the ML models we implemented in the PCCI approach.

After implementing ML models using the MATLAB platform, we used the same dataset, but with different models. Table 2 shows the accuracy results for eight models from various algorithms.

The implementation of the data is performed by dividing the data into features to predict the methods of attacks, x, and the predicted values, the methods, y. After that, both x and y are divided into training and testing sets to perform the SVM algorithm on them. The result of testing the model is printed in confusion matrix with the accuracy of the model. A more profound comprehension of data and analytical models based on ML that uses a large amount of cybersecurity data can be adequate to accomplish the objective. Additionally, modifying the data and relevant techniques could increase the performance of the resulting prediction model and improve its suitability for a cybersecurity domain. In this experiment, we used MATLAB and Python to implement the prediction. Python has a general programming language and various ML libraries, such as (NumPy and SciPy). However, ML algorithms in MATLAB use computational methods to acquire information from data without relying on a fixed equation. Both have different ways of using and presenting the results in different styles.

In general, supervised and unsupervised learning are ML algorithms. A computer program is trained using a set of sample inputs and the expected outputs in supervised learning. ML models have been used in the PCCI approach. When the outcomes are discrete, this type of ML can be applied to classification, and when the outcomes are continuous, it can be applied to regression. However, unsupervised learning uses unlabeled data as inputs without related output variables. This algorithm aims to find patterns or structures in a large dataset and to reduce the number of variables.

On the other hand, Logistic Regression and Decision Tree Classifiers are used in Python. Performing experiments on ML models show accuracy results between 66.25% for the linear discriminant model, the highest accuracy, and 57.2% fine tree, the lowest accuracy results. The highest model, the linear discriminant model, considers a linear relationship between the target variable and the features. One of the objectives of this approach is to develop the best model that predicts cyberattacks with high accuracy. If we want to use a case study for this approach, it depends on the dataset that we need to obtain from this organization. Table 3 shows a summary and the robustness of each ML algorithm used in the different industrial sectors, the attacker motivations, and attack methods.

Enabling cyberattack prediction will encourage the creation of many services and products, resulting in a massive and active market. A fine tree or linear discriminant model can be used as a simple model. The PCCI approach can be implemented in any CI organization that provides the required dataset to run the PCCI approach [46].

## 5. Conclusions

The increasing connectivity and digitization of current CI systems have led to improvements in efficiency, productivity, cost, and quality. However, this connectivity also poses potential risks related to digitalization, generating more data, and increasing connectivity. To address these risks, it is essential to design appropriate security solutions for CIs. This paper proposes a novel approach to predicting cyberattacks at an early stage through a proactive approach to identifying CI security threats. The core of the approach is the dataset, which is used to ensure prediction accuracy by minimizing false-positive alerts. The approach uses ML techniques to train the dataset and predict cyberattacks based on real cyber incident data from various CI sectors. The prediction mechanism and models depend on factors such as attacker and adversary motivations and the nature of the CI. The accuracy of the approach relies on the dataset, which can be improved by incorporating more data. This approach can provide executive management and security specialists with valuable insights and information to prioritize security countermeasures.

## Figures and Tables

**Table 1:** Performance Results.

Classifier	Accuracy	Precision	Recall	F1-Score
Linear	66.25%	18.5%	36%	13%
Logistic Regression	58.5%	15.6%	12.4%	10.4%
Decision Tree Classifier	60%	46.5%	41.1%	41.7%
Poly	65%	7.2%	11.1%	8.7%

**Table 2:** Performance Results.

Classifier	Accuracy	Precision	Recall	F1-Score
Fine Tree	57.2%	51.4%	77.0%	61.64%
Quadratic SVM	64.8%	57.0%	91.0%	70.09%
Linear Discrimination	59.5%	70.7%	94.8%	80.99%
Fine Gaussian SVM	64.2%	72.8%	95.7%	82.69%

**Table 3:** Summaries of the impact level for ML algorithm, attack methods, and CI sector.

Impact Level	ML Classifiers	Attack Methods	CI Sector
Highest	Linear	Malware	Civil administration
Medium	Decision tree	DDOS attack	Energy
Lowest	Fine tree	Jamming and spoofing	Water

## Data Availability

Not applicable.
